# Changes in health risk behaviors of elementary school students in northern Taiwan from 2001 to 2003: results from the child and adolescent behaviors in long-term evolution study

**DOI:** 10.1186/1471-2458-7-323

**Published:** 2007-11-12

**Authors:** Wen-Chi Wu, Hsing-Yi Chang, Lee-Lan Yen, Tony Szu-Hsien Lee

**Affiliations:** 1 Center for Health Policy Research and Development, National Health Research Institutes. No. 35, Keyan Road, Zhunan Town, Miaoli County 350, Taiwan, R.O.C; 2 Institute of Health Policy and Management, College of Public Health, National Taiwan University. Rm. 623, No. 17, Xu-Zhou Road, Taipei 100, Taiwan, R.O.C; 3 Department of Health Education, National Taiwan Normal University. 162 Sec. 1 Ho-Ping East Road, Taipei 106, Taiwan, R.O.C

## Abstract

**Background:**

Previous research has indicated that children's behaviors have long-term effects on later life. Hence it is important to monitor the development of health risk behaviors in childhood. This study examined the changes in health risk behaviors in fourth- to sixth-grade students in northern Taiwan from 2001 to 2003.

**Methods:**

The Child and Adolescent Behaviors in Long-Term Evolution (CABLE) study collected data from 1,820 students from 2001 to 2003 (students were 9 or 10 years old in 2001). Exploratory factor analysis was used to determine the aggregation of health risk behaviors. A linear growth curve model was used to determine whether health risk behaviors changed over time.

**Results:**

Of the 13 behaviors, staying up late and eating snacks late at night were the most prevalent (82.3% of subjects in 2001, 81.8% in 2002, 88.5% in 2003) and second most prevalent (68.7%, 67.4%, 71.6%) behaviors, respectively, from 2001 to 2003. The three least prevalent health risk behaviors were chewing betel nut (1.0%, 0.4%, 0.2%), smoking (1.4%, 1.0%, 0.8%), and drinking alcohol (8.5%, 6.0%, 5.2%). The frequencies of swearing and staying up late showed the greatest significant increases with time. On the other hand, suppressing urination and drinking alcohol decreased over time. Using exploratory factor analysis, we aggregated the health risk behaviors into three categories: unhealthy habits, aggressive behaviors, and substance use. Although students did not display high levels of aggressive behavior or experimentation with substances, the development of these behaviors in a small proportion of students should not be ignored. The results of the linear growth curve model indicated that unhealthy habits and aggressive behaviors increased over time. However, substance use slightly decreased over time.

**Conclusion:**

We found that some health risk behaviors increased with time while others did not. Unhealthy habits and aggressive behaviors increased, whereas substance use slightly decreased during this period. Educational professionals should pay attention to the different patterns of change in these behaviors in elementary school students.

## Background

Health risk behaviors such as smoking, alcohol abuse, unhealthy dietary patterns, sedentary lifestyle, unsafe behaviors, and aggressive behaviors have been found to have an important influence on morbidity and mortality [1-3]. These behaviors not only influence individuals' health but also create burdens for the nation and society as a whole. It is well documented that behaviors developed during childhood influence health in adolescence and adulthood [4-6]. Furthermore, one longitudinal study reported that behaviors of seventh graders, including physical activity, food preference behaviors, and smoking, consolidated early [7]. Thus, helping children establish healthy lifestyles and avoid developing health risk behaviors is crucial and should be started before these behaviors are firmly established. In other words, because children's behaviors have long-term effects on later life, it is important to monitor the development of children's health risk behaviors earlier, in order to design health promotion programs for children.

Health risk behaviors, which develop over time, often correlate with each other [8-11]. Some researchers have further found that people with multiple risk behaviors are at greater risk of developing chronic diseases and suffering injuries than those with only one risk behavior [12,13]. However, very few researchers have monitored the development of aggregated risk behaviors. Cohort studies can assess relationships among the outcomes and variables under study, many of which may be temporal in nature, thus leading to causal models. However, longitudinal research is often difficult to conduct and adequate statistical methods have been lacking. Time trends of the different types of risk behaviors starting from early childhood have rarely been reported. Nevertheless, longitudinal studies provide essential information about the development of different types of risk behaviors [14]. Recent developments in multilevel analysis of hierarchical data in longitudinal studies have provided efficient methods to estimate the change over time [14,15]. We are fortunate to have developed a student cohort. The main purpose of this study was to determine whether health risk behaviors of elementary school students changed from 2001 to 2003.

## Methods

### Study sample

Data were extracted from the Child and Adolescent Behaviors in Long-Term Evolution (CABLE) study [16], which was initiated in 2001. Samples for the CABLE study were chosen from 9 public elementary schools in Taipei City (representing a metropolitan area) and 9 in Hsinchu County (representing a rural area). The schools were selected by randomized cluster sampling based on school size. There were 152 elementary schools in Taipei City and 79 in Hsinchu County in 2000. Based on the number of students, schools were divided into small, medium-sized, and large. Six small schools, 2 medium-sized schools, and 1 large school were selected from each location. The details of CABLE's study design and sampling method have been reported previously [11,16].

The CABLE study was approved by the international review board of the National Health Research Institutes in Taiwan. All parents of students in the study were asked to sign a consent form if their children agreed to participate in the study [16]. The CABLE cohort consisted of 2,075 fourth graders in 2001. We analyzed data on the 1,820 sixth-grade students in 2003 who had completed the previous two surveys. In other words, we followed participants from age 9 or 10 years (fourth grade) to age 11 or 12 years (sixth grade). The follow-up rate was 87.7%. Losses to follow up were due to absence from school due to illness, transferring to another school, school reorganization and refusal to participate. There were no significant differences between the original sample and the sample who completed follow up in demographic characteristics. Hence these losses are likely to be random in nature and should not have significantly biased the results. The study sample included 943 boys (51.81%) and 877 girls (48.19%); 965 (53.02%) were from Taipei City and 855 (46.98%) were from Hsinchu County.

### Study variables

The 13 health risk behaviors of interest to us in our study included (1) staying up late (past 10:00 p.m.), (2) eating snacks late at night (that is, before sleep), (3) eating fast food (such as carry-out lunches, fried chicken, and hamburgers), (4) suppressing urination (having the urge to urinate but not passing urine), (5) playing video games for prolonged periods (more than 2 consecutive hours), and (6) watching television for prolonged periods (more than 2 consecutive hours). In addition, the following behaviors were also considered: (7) swearing, (8) throwing things when angry, (9) hitting others, (10) vandalism, (11) smoking, (12) drinking alcohol and (13) chewing betel nut. We asked students to report occurrences of health risk behaviors 1 through 6 that they had engaged in during the week before the interview, and occurrences of the remaining 7 health risk behaviors engaged in during the month before the interview. Behavioral performance was measured using a four-point scale: never, once or twice, many times, and almost every day. (Additional file 1.)

Most of these behaviors were selected based on previous studies [17-20], such as watching TV, playing video games, hitting others, smoking and drinking alcohol, and one behavior instrument [21], such as lack of sleep, swearing, throwing things, and vandalism. Eating fast food and late night snacks were included because they are both related to childhood obesity in Taiwan and Japan [22,23]. Suppressing urination was included as due to the traditional educational style in Taiwan; some students do not ask permission to go to the bathroom during class as they are embarrassed about raising their hands. This suppression of urination may be detrimental to their bladder. Chewing betel nut is a prevalent local custom in Taiwan and was selected based on its relationship with oral cancer [24]. All health risk behaviors were related to health outcomes, not only physical but also mental and social. For example, swearing, one of the verbal aggressive behaviors, is related to self-concept damage, hurt feelings, relational deterioration and even physical aggression [25]. Hence, swearing can be harmful to mental and social health. Staying up late could lead to lack of sleep and result in externalizing behaviors and attention and social problems [26]. Particular health outcomes may result from more than one risk behavior. For example, eating fast food, short sleep duration, and watching TV for long periods are all related to child overweight [23]. The CABLE study questionnaire has been carefully refined for reliability and validity [11,16]. Ten experts, including a psychologist, sociologist, behavioral scientist, health educator, and elementary school teacher, were invited to give suggestions on improving validity. A pilot study was conducted on 84 fourth graders to ensure that wording was appropriate. The validity and reliability of the questionnaire were also analyzed using pilot data.

### Data analysis

Percentages of students with health risk behaviors were calculated for each year by adding up percentages for the occurrences once or twice, many times, and almost every day. Behaviors were then ranked from highest to lowest prevalence for each year. Linear trend analysis was used to determine whether each behavior varied with time.

Exploratory factor analysis was carried out to examine aggregating characteristics of the 13 health risk behaviors based on 2001 data [27]. The statistical software SAS 8.02 was used to perform exploratory factor analysis using principal component analysis and varimax rotation to estimate the pattern coefficient [28]. If the latent structure of health risk behaviors can be demonstrated, we can conclude that the health risk behaviors can be aggregated in childhood. Different latent factors extracted through factor analysis indicated different types of health risk behaviors.

To confirm the results of the factor analysis, we added up the original points of the behaviors under each factor to derive total scores for each student over the 3 year period. All items were assigned 0 to 3 points indicating never, once or twice, many times, and almost every day. Higher scores in particular factors indicated that the student performed higher levels of this particular type of risk behavior in that year. We used these scores of different types of health risk behaviors as the dependent variable in the following longitudinal analysis.

Considering the variances between individuals and time points, we analyzed the longitudinal data using a linear growth curve model [29-31]. The statistical software MLwiN 2.0 was used [32]. We use iterative generalized least squares to estimate parameters after applying the linearization. Because the study sample structure consisted of measurements of 3 years nested within students, the units of this research sample lay at two hierarchical levels. (Actually, the students were nested within schools. However, the variances in school level were not statistical significant; hence we omitted the differences in school level.) We modeled a two-level random intercept model to fit measures repeating three times. A time variable was included in the model to examine the time trends for health risk behaviors. A positive parameter indicated that the particular type of behavior increased over time.

The statistical model was as follows:

*Y*_ij _= *β*_0 _+ *β*_1_Time + *u*_0j _+ *e*_ij_

where *i *represents the time point, *j *represents the study subject, *Y*_ij _represents the level of expression of the health risk behavior by subject *j *at time *i*, *β*_0 _is the total mean, *β*_1 _is the coefficient for time trend of the risk behaviors over time, *u*_0j _is the variation between students, and *e*_ij _is the variation within students. *β*_0 _and *β*_1 _are fixed effects and do not vary between individuals or over time; *u*_0j _and *e*_ij _are random effects and vary between individuals or over time. Furthermore, in the multiple regression models, we added residential area and gender as control variables.

## Results

### Distribution and time trend of each health risk behavior in study participants

Table 1 shows the distribution of health risk behaviors in the subjects from 2001 (grade 4) to 2003 (grade 6). Percentages of students ever having health risk behaviors were calculated by adding up percentages of the occurrences once or twice, many times, and almost every day. The most prevalent and second most prevalent health risk behaviors for each year were staying up late and eating snacks late at night, respectively. In 2001, the third most prevalent behavior was watching television for prolonged periods. In 2002, the third most prevalent behavior was eating fast food, and in 2003, it was swearing. The two least prevalent behaviors over the 3-year period were chewing betel nut and smoking. Compared to the smoking rate (over 20% of 9th to 12th graders in the US smoked in the 30 days preceding the survey in 2001 and 2003) reported by YRBS [33], in this sample fewer than 1% of 4th to 6th graders smoked in the same period. However, further follow up is required to see whether the smoking rate will catch up to the rate in the US when the students reach high school.

**Table 1:** Distribution of health risk behaviors in subjects by grade (*n *= 1,820)

Behavior	Grade 4 (2001)					Grade 5 (2002)					Grade 6 (2003)				
	once/twice	many times	every day	**ever**	**Rank**	once/twice	many times	every day	**ever**	**Rank**	once/twice	many times	every day	**ever**	**Rank**
Staying up late	45.13	22.43	14.73	**82.29**	**1**	45.19	20.89	15.67	**81.75**	**1**	39.82	29.59	19.03	**88.44**	**1**
Eating snacks at night	45.38	13.63	9.73	**68.74**	**2**	46.43	11.76	9.18	**67.37**	**2**	47.28	15.52	8.75	**71.55**	**2**
Watching TV for prolonged periods	36.73	19.83	11.58	**68.14**	**3**	33.02	17.78	10.18	**60.98**	**4**	36.03	20.74	12.38	**69.15**	**4**
Eating fast food	56.77	5.94	1.82	**64.53**	**4**	57.64	5.00	1.32	**63.96**	**3**	54.87	5.72	1.10	**61.69**	**5**
Swearing	46.37	9.02	3.69	**59.08**	**5**	46.08	10.33	4.03	**60.44**	**5**	48.16	17.01	6.05	**71.22**	**3**
Hitting others	43.53	8.98	1.82	**54.33**	**6**	31.98	4.29	1.98	**38.25**	**7**	39.45	7.53	2.47	**49.45**	**6**
Suppressing urination	44.56	7.01	2.43	**54.00**	**7**	41.26	3.41	1.48	**46.15**	**6**	41.42	5.23	0.94	**47.59**	**7**
Playing video games for prolonged periods	29.04	8.26	5.95	**43.25**	**8**	21.37	6.77	6.17	**34.31**	**8**	22.78	12.30	9.05	**44.13**	**8**
Throwing things when angry	23.19	2.65	1.82	**27.66**	**9**	18.02	2.7	1.32	**22.04**	**9**	22.16	3.52	1.59	**27.27**	**9**
Drinking alcohol	7.2	0.93	0.33	**8.46**	**10**	5.39	0.55	0.06	**6.00**	**11**	4.18	0.66	0.38	**5.22**	**11**
Vandalism	6.35	0.55	0.17	**7.07**	**11**	6.27	0.66	0.33	**7.26**	**10**	7.20	0.88	0.22	**8.30**	**10**
Smoking	1.16	0.17	0.06	**1.39**	**12**	0.82	0.11	0.05	**0.98**	**12**	0.71	0.11	0.00	**0.82**	**12**
Chewing betel nut	0.55	0.33	0.11	**0.99**	**13**	0.33	0.00	0.05	**0.38**	**13**	0.11	0.05	0.00	**0.16**	**13**

According to the results of linear trend analysis, behaviors that increased significantly over the 3-year period were swearing, staying up late and playing video games for prolonged periods (Table 2). The behaviors are ranked by t-value to show the extent of increasing or decreasing with time. The behavior with the largest increase was swearing (t value is 11.87). These behaviors showed identical trends across gender. Behaviors that decreased significantly were suppressing urination, drinking alcohol, chewing betel nut, hitting others and eating fast food. However, for girls, there were no linear trends for declines in drinking alcohol, chewing betel nut, hitting others and eating fast food. Among boys, eating snacks at night increased and smoking decreased. Among girls, both vandalism and watching TV increased. Therefore, it can be seen that some behaviors showed different time trends across gender. Nevertheless, the mean score of each health risk behavior was still higher for boys than girls.

**Table 2:** Time trends of health risk behaviors in subjects by grade (*n *= 1,820)

Behaviors	Total								Boys								Girls							
			
	Grade 4 (2001)		Grade 5 (2002)		Grade 6 (2003)		Linear trend analysis		Grade 4 (2001)		Grade 5 (2002)		Grade 6 (2003)		Linear trend analysis		Grade 4 (2001)		Grade 5 (2002)		Grade 6 (2003)		Linear trend analysis	
	
	Mean	SD	Mean	SD	Mean	SD	T value	Sig.	Mean	SD	Mean	SD	Mean	SD	T value	Sig.	Mean	SD	Mean	SD	Mean	SD	T value	Sig.
1. Swearing	1.75	0.77	1.79	0.78	2.00	0.84	11.87	***	1.86	0.81	1.91	0.83	2.11	0.83	8.56	***	1.65	0.71	1.66	0.71	1.89	0.83	8.24	***
2. Staying up late	2.34	0.93	2.34	0.95	2.56	0.93	8.56	***	2.38	0.95	2.38	0.99	2.57	0.93	5.18	***	2.30	0.92	2.29	0.90	2.55	0.92	7.03	***
3. Playing video games for prolonged periods	1.63	0.87	1.53	0.87	1.75	0.99	4.61	***	1.86	0.97	1.80	1.00	1.99	1.08	3.30	**	1.39	0.66	1.25	0.57	1.48	0.80	3.29	**
4. Vandalism	1.08	0.31	1.09	0.33	1.10	0.34	1.67	-	1.12	0.38	1.12	0.40	1.13	0.39	0.34	-	1.04	0.19	1.05	0.24	1.06	0.28	2.68	**
5. Watching TV for prolonged periods	2.11	0.98	1.99	0.99	2.15	1.00	1.35	-	2.18	1.02	2.05	1.04	2.17	1.02	-0.28	-	2.04	0.94	1.92	0.93	2.12	0.97	2.36	*
6. Eating snacks at night	2.02	0.92	1.97	0.90	2.05	0.89	1.10	-	2.01	0.94	2.03	0.94	2.09	0.92	2.14	*	2.03	0.89	1.92	0.85	2.00	0.85	-0.71	-
7. Throwing things when angry	1.34	0.62	1.27	0.58	1.34	0.62	-0.01	-	1.40	0.68	1.33	0.64	1.39	0.67	-0.57	-	1.28	0.55	1.22	0.50	1.29	0.57	0.73	-
8. Smoking	1.02	0.15	1.01	0.13	1.01	0.11	-1.77	-	1.03	0.20	1.02	0.17	1.01	0.12	-2.28	*	1.00	0.08	1.00	0.07	1.01	0.09	0.61	-
9. Eating fast food	1.74	0.65	1.72	0.62	1.70	0.63	-2.42	*	1.77	0.67	1.74	0.64	1.73	0.65	-1.61	-	1.71	0.63	1.69	0.59	1.66	0.59	-1.85	-
10. Hitting others	1.67	0.71	1.46	0.67	1.62	0.73	-2.47	*	1.72	0.74	1.46	0.67	1.58	0.72	-4.76	***	1.61	0.68	1.47	0.68	1.66	0.75	1.45	-
11. Chewing betel nut	1.02	0.17	1.00	0.09	1.00	0.06	-3.57	***	1.03	0.22	1.01	0.12	1.00	0.08	-3.14	**	1.00	0.08	1.00	0.03	1.00	0.00	-1.85	-
12. Drinking alcohol	1.10	0.36	1.07	0.28	1.07	0.31	-3.62	***	1.13	0.41	1.09	0.33	1.07	0.34	-3.95	***	1.07	0.28	1.04	0.20	1.06	0.28	-0.78	-
13. Suppressing urination	1.66	0.71	1.53	0.64	1.55	0.64	-5.94	***	1.65	0.74	1.50	0.62	1.52	0.63	-4.77	***	1.67	0.69	1.55	0.65	1.58	0.65	-3.57	***

*: p < 0.05, **: p < 0.01, ***: *P *< 0.001.

The frequencies of some behaviors decreased in the second year, namely 5^th ^grade, such as playing video games for prolonged periods, watching TV for prolonged periods, eating snacks at night, throwing things when angry, and hitting others. Possible reasons for this are discussed further below.

### Latent structure of health risk behaviors

Exploratory factor analysis was used to look at the general patterns of the 13 behaviors and to extract the latent structure of subjects' health risk behaviors in 2001 (grade 4), as shown in Table 3. Using an eigenvalue > 1 as the criterion, the 13 behaviors could be grouped into three factors: (1) aggressive behaviors (swearing, throwing things when angry, hitting others, and vandalism), (2) substance use behaviors (smoking, drinking alcohol, and chewing betel nut), and (3) unhealthy habits (staying up late, eating snacks late at night, eating fast food, suppressing urination, playing video games for prolonged periods, and watching television for prolonged periods). The percentage of variance explained by these three factors was more than 40%. The behaviors under each factor may be affected by some similar correlates. Further, we analyzed the factor structures of these behaviors separately for rural and urban areas. The data showed no differences in patterns between these areas. Hence, we can say that the health risk behaviors can be aggregated into three types for the period beginning when the students were in the fourth grade.

**Table 3:** Rotated factor structure of health risk behaviors of subjects in grade 4 in 2001

	Factor 1	Factor 2	Factor 3
	
Behavior	aggressive behaviors	substance use behaviors	unhealthy habits
Hitting others	**0.751**	0.006	0.081
Swearing	**0.702**	0.000	0.057
Throwing things when angry	**0.645**	0.118	0.190
Vandalism	**0.434**	0.215	0.026
Smoking	0.066	**0.829**	0.017
Chewing betel nut	0.021	**0.797**	0.017
Drinking alcohol	0.149	**0.678**	0.070
Eating fast food	0.065	--0.009	**0.583**
Eating snacks at night	--0.045	0.055	**0.578**
Staying up late	--0.030	0.033	**0.544**
Watching TV for prolonged periods	0.290	0.003	**0.520**
Playing video games for prolonged periods	0.295	0.100	**0.498**
Suppressing urination	0.309	--0.041	**0.393**

Eigenvalue	2.689	1.682	1.141
Explained variance (%)	0.207	0.129	0.088
Cumulative explained variance (%)	0.207	0.336	0.424
Cronbach's α	0.5935	0.6638	0.5200

KMO measure of sampling adequacy = 0.744.

### Description and longitudinal trends of aggregated health risk behaviors

To analyze longitudinal trends in the aggregated behaviors, we calculated the total scores for each type of health risk behavior by adding up the points for behaviors under each factor. Participants' patterns of aggregated health risk behaviors from 2001 (grade 4) to 2003 (grade 6) are shown in Table 4. The range of possible points for aggressive behaviors was 4–16, that for substance use behaviors was 3–12, and that for unhealthy habits was 6–24. Generally, the means of aggressive behaviors and unhealthy habits increased from 2001 to 2003, with a slight decrease in the second year. Substance use behaviors decreased slightly from 2001 to 2003. Given the range of possible points for these three types of behaviors, the means indicate that the students in this study exhibited mild or moderate health risk behaviors.

**Table 4:** Means and standard deviations of aggregated health risk behaviors over 3 years

	Aggressive behaviors			Substance use behaviors			Unhealthy habits		
	
	Grade 4	Grade 5	Grade 6	Grade 4	Grade 5	Grade 6	Grade 4	Grade 5	Grade 6
Mean	5.85	5.61	6.06	3.13	3.08	3.08	11.50	11.08	11.74
Standard deviation	1.70	1.66	1.79	0.54	0.38	0.36	2.77	2.83	2.87

The results of the linear growth curve model are shown in Table 5. Analysis was carried out after adding the time variable [32], so that any changes in the performance of health risk behaviors over time could be confirmed. The results show that the coefficients of time for unhealthy habits and aggressive behaviors were positive, meaning that these types of health risk behaviors increased over time. The coefficient of time for substance use behaviors was small and negative, meaning that this type of behavior decreased slightly over time. Comparing model 2 to model 1, the effect of time was unchanged after controlling for sex and location. Boys expressed more health risk behavior than girls for all three types of behavior and students who lived in Hsinchu (rural) expressed more health risk behaviors than those who lived in Taipei (urban).

**Table 5:** Time trends of aggregated health risk behaviors of subjects from grade 4 to grade 6

	Unhealthy habits				Aggressive behavior				Substance using behavior			
	Model 1		Model 2		Model 1		Model 2		Model 1		Model 2	
Fixed effects	Coef.	SD.	Coef.	SD.	Coef.	SD.	Coef.	SD.	Coef.	SD.	Coef.	SD.
Intercept	10.680*	0.113	10.676*	0.113	5.321*	0.069	5.321*	0.069	3.097*	0.017	3.097*	0.017
Time	0.119*	0.036	0.119*	0.036	0.104*	0.022	0.104*	0.022	-0.027*	0.006	-0.027*	0.006
Boy vs. Girl			0.758*	0.102			0.419*	0.062			0.072*	0.014
Hsinchu vs. Taipei			0.287*	0.102			0.194*	0.062			0.037*	0.014
												
Random effects	Var.	SD.	Var.	SD.	Var.	SD.	Var.	SD.	Var.	SD.	Var.	SD.

Individual variability	3.156*	0.161	3.156*	0.161	1.135*	0.059	1.135*	0.059	0.039*	0.003	0.039*	0.003
Time variability	4.709*	0.110	4.709*	0.110	1.789*	0.042	1.789*	0.042	0.146*	0.003	0.146*	0.003

* p: < 0.05

## Discussion

We observed changes in health risk behaviors of school children in Taiwan from 2001 to 2003. In terms of single behaviors, behaviors that increased with time were swearing, staying up late, and playing video games for prolonged periods, and behaviors that decreased with time were suppressing urination, drinking alcohol, chewing betel nut, hitting others and eating fast food. As a whole, the 13 health risk behaviors in elementary school students could be aggregated into three latent factors: unhealthy habits, aggressive behaviors, and substance use behaviors. The results are similar to findings in previous studies [8-13]. After controlling for residential area and gender, we found that unhealthy habits and aggressive behaviors increased from 2001 to 2003, but that substance use behaviors decreased slightly.

The two leading health risk behaviors for students since fourth grade were staying up late and eating snacks at night (Table 1). The least prevalent health risk behavior was chewing betel nut. The prevalences of these behaviors were relatively stable over the 3 years. However, the aggressive behavior of swearing jumped from being the fifth to being the third most common behavior from 2001 to 2003. The result of time trend analysis also showed that the frequency of swearing had the most significant increase with time. Compared to the rate of seeing other 13–15 year olds swearing, insulting or making nasty comments at least once or twice a month reported by a research group in the US [34], the frequency of swearing in this study sample (59.08%~71.22%) was higher than the US sample (14%~18%). Even though the participants in the US were older and reported what they observed rather than their own situation, the rate in our sample was almost three-fold that in the US. Therefore, grade 5 could be the appropriate time for preventive education targeting aggressive behaviors, especially verbal aggression in Northern Taiwan.

It is important not to ignore substance use behaviors, as once they have developed they can be difficult to break. When looking at individual behavioral items, two of the three substance use behaviors decreased from 2001 to 2003 (Table 5). The total score of aggregated substance use behaviors also decreased slightly. We used stricter definitions (student took one puff, took one sip of alcohol, and chewed one piece of betel nut) for substance use behaviors. We found that more children had drunk alcohol than smoked or chewed betel nut (Table 1). One possible reason is that during celebrations or feasts in Taiwan, parents often let children have a taste of alcohol. Although our study did not find high rates of substance use behaviors, the results still indicate that a small proportion of children have tried using these substances at a young age. Furthermore, the decrease in each substance use behavior was only found among boys, and girls did not show the same trends (Table 2). Research indicates that gender differences exist in health-related beliefs and health behavior [35-37]. We found that boys and girls had different substance use behavior patterns. Although boys engaged in substance use more frequently than girls, girls did not show the same pattern of decline as boys. Hence, attention still needs to be given to girls' substance using behaviors.

The three types of health risk behaviors showed different time trends. Unhealthy behavior and aggressive behavior increased with time, whereas substance use behavior decreased with time (Table 5). However, comparing the results of time trend analysis for the aggregated behaviors to those for single behaviors (Table 2), we can see that not all of the single behaviors followed the general time pattern. Staying up late and playing video games for prolonged periods showed the greatest contribution to the increasing pattern of unhealthy behavior. Likewise, swearing played a major role in the increase in aggressive behavior. For substance use behavior, there was more consistency between changes in single behaviors and the total score. From these results we can see that although a health risk behavior type may have a general time pattern as a whole, each single behavior may still have its own unique time pattern. For example, for the six behaviors that were grouped as unhealthy habits, the first behavior to increase could be staying up late. For aggressive behavior, swearing may be the first behavior in this category. Therefore, as health educators, although we can develop strategies targeting groups of related behaviors, we still need to pay attention to the different development time courses of each behavior in the group. As a result, two different strategies would be necessary for health education, namely those focusing on single behaviors as well as those focused on groups of behaviors.

The mean scores of aggressive behaviors and substance use behaviors suggest that these types of behaviors were quite infrequent and that only a small proportion of students experiment with substances at this young age (Table 3). This finding is probably due to the fact that the participants came from the general population. However, Millstein et al. [38] indicated that an extremely important and currently neglected area in professional education is knowledge about the general adolescent population. Our study is one of the few studies to fill this gap.

Furthermore, we unexpectedly found that some behaviors decreased in the 5^th ^grade, as well as the mean scores of the three behavioral groups. This is probably because students in Taiwan are rearranged into new classes in the 5th grade and therefore, most of the students will be with new classmates at this time. They are too unfamiliar with each other to undertake risk behaviors together such as playing video games, throwing things when angry, and hitting others. They may also feel too stressed to relax on their own by doing things such as watching TV, eating snacks late at night and fast food. However, we have no evidence to support this hypothesis and further research is needed to solve this puzzle.

Laaksonen et al. [13] showed that having three or four health risk behaviors concurrently is related to age: having such behaviors was most common in those aged 20–34, less common in those aged 35–49, and least common in those aged 50–64. According to our results, health risk behaviors are engaged in as early as elementary school. Millstein et al. [39] suggested that we should stop viewing young adolescents as naive children and begin to view them as participants in a changing social environment. As a result, they should be educated about healthy behaviors and encouraged to develop healthy behaviors in childhood.

As shown in Table 5, substance use behaviors slightly decreased over time. The decrease in substance use behaviors is possibly due to implementation of anti-smoking legislation and education with an emphasis on drugs and smoking. Such legislation and education have strengthened the formation of negative social norms and restrictive attitudes about these kinds of behaviors. One possible reason for the increase in unhealthy habits and aggressive behaviors over time is maturity. As students move from grade 4 to grade 6, not only are their bodies developing and maturing, but they are also undergoing psychological development. As these students become more independent and develop a stronger sense of self, they are less willing to accept the restrictions placed on them by parents and schools. In addition, they are influenced by the media, television programs, and computer and video games. The increase in staying up late is likely due to greater amounts of homework, and the reason for the increase in swearing could be peer pressure or the increasing acceptability of swearwords. This project will continue to collect information on the students to learn more about the time trends of health risk behaviors and their effects.

The participants in this study represent only students of public schools in Taipei City and Hsinchu County in northern Taiwan. In addition, the study focused on health risk behaviors over only 3 years. These limitations restrict the generalizability of our findings. We suggest that future studies involve different populations and monitor behaviors for longer durations to more closely examine the issue of health risk behaviors in childhood.

## Conclusion

We found that some health risk behaviors (swearing, staying up late and playing video games for prolonged periods) increased with time, while some others (suppressing urination, drinking alcohol, chewing betel nut, hitting others and eating fast food) did not decrease or stayed stable. In general, health risk behaviors of children can be aggregated into three types; unhealthy habits, aggressive behaviors and substance use. Although the frequency of aggressive behaviors and substance use was low, after controlling for gender and area of residence, the frequencies of unhealthy habits and aggressive behaviors increased significantly from 2001 to 2003, whereas substance use slightly decreased. Educational professionals should pay attention to the different patterns of health risk behaviors in elementary school students and preventive measures for behaviors that increase during this period should be initiated earlier in childhood.

## Competing interests

The author(s) declare that they have no competing interests.

## Authors' contributions

WCW wrote the paper and conducted statistical analyses. HYC revised the article and gave statistical advice. LLY contributed to the study design and led the CABLE research team. TSHL made suggestions regarding the Discussion section. All authors read and approved the final manuscript.

## Pre-publication history

The pre-publication history for this paper can be accessed here:



## Supplementary Material

Additional file 1 Appendix A. Wording of health risk behaviors in the questionnaire. The appendix shows the wording and the scales for measuring the 13 health risk behaviors. Click here for file
