# Supplementary material for: Changes in health risk behaviors of elementary school students in northern Taiwan from 2001 to 2003: results from the child and adolescent behaviors in long-term evolution study
Source: BMC Public Health. 2007 Nov 12;7:323. doi: 10.1186/1471-2458-7-323 (PMC2198917; doi:10.1186/1471-2458-7-323)
Supplement: Additional file 1 — Appendix A. Wording of health risk behaviors in the questionnaire. The appendix shows the wording and the scales for measuring the 13 health risk behaviors. [file 1471-2458-7-323-S1.doc]

Appendix A. Wording of health risk behaviors in the questionnaire.

| Health risk behavior | Wording of question |
| --- | --- |
| Eating fast food | Did you eat fast food such as hamburgers, french fries, fried chicken, instant noodles, or pizza last week? |
| Eating snacks at night | Did you eat snacks before sleep such as milk, fruit, or sweets last week? |
| Staying up late | Did you go to bed later than 10 p.m. last week? |
| Watching TV for prolonged periods | Did you watch television for more than two hours straight last week? |
| Playing video games for prolonged periods | Did you play computer or video games for more than two hours straight last week? |
| Suppressing urination | Did you ignore the urge to urinate last week? |
| Hitting others | Did you hit anyone last month? |
| Swearing | Did you swear at anyone last month? |
| Throwing things when angry | Did you throw things when you were angry last month? |
| Vandalism | Did you vandalize anything at school last month? |
| Smoking | Have you ever smoked (even one puff)? |
| Chewing betel nut | Have you ever chewed betel nut (even one piece)? |
| Drinking alcohol | Have you ever used alcohol (even one sip)? |

The scales for measuring hitting others, swearing, throwing things when angry, vandalism, smoking, drinking alcohol, and chewing betel nut were: 1= never; 2=once or twice last month; 3= several times last month; 4=every day last month. The scales for measuring the rest of the health risk behaviors were: 1=never; 2=once or twice last week; 3=several times last week; 4=every day last week.
